# Correlations between Salivary Immuno-Biochemical Markers and HbA1c in Type 2 Diabetes Subjects before and after Dental Extraction

**DOI:** 10.3390/antiox10111741

**Published:** 2021-10-30

**Authors:** George-Alexandru Maftei, Maria-Alexandra Martu, Marius-Cristian Martu, Dora Popescu, Petra Surlin, Diana Tatarciuc, Cristina Popa, Liliana-Georgeta Foia

**Affiliations:** 1 Department of Oral Medicine, Grigore T. Popa University of Medicine and Pharmacy, 16 Universitatii Str., 700115 Iasi, Romania; george.maftei.gm@gmail.com (G.-A.M.); cristina.popa@umfiasi.ro (C.P.); 2 Department of Periodontology, Grigore T. Popa University of Medicine and Pharmacy, 16 Universitatii Str., 700115 Iasi, Romania; 3 Department of ENT, Grigore T. Popa University of Medicine and Pharmacy, 16 Universitatii Str., 700115 Iasi, Romania; 4 Department of Periodontology, University of Medicine and Pharmacy of Craiova, 2 Petru Rares Str., 200349 Craiova, Romania; dora.popescu@umfcv.ro (D.P.); petra.surlin@umfcv.ro (P.S.); 5 Department of Internal Medicine, Faculty of Medicine, Grigore T. Popa University of Medicine and Pharmacy, 16 Universitatii Str., 700115 Iasi, Romania; diana.tatarciuc@gmail.com; 6 Department of Biochemistry, Grigore T. Popa University of Medicine and Pharmacy, 16 Universitatii Str., 700115 Iasi, Romania; georgeta.foia@umfiasi.ro

**Keywords:** oxidative stress, salivary diagnosis, dental extraction, diabetes mellitus

## Abstract

Dental extraction can trigger certain sequences of complex processes that involve both hard (alveolar bone) and soft tissue (periodontal ligament, gingiva) remodeling. Type 2 diabetes is a serious risk factor for many oral pathologies, both in terms of progression and severity, but also regarding subsequent rehabilitation possibilities. The aim of this study was to establish whether certain molecules: osteoprotegerin (OPG), kappa B nuclear factor receptor activator ligand (RANKL), hepatocyte growth factor (HGF), tumor necrosis factor-α (TNF-α), interleukin 18 (IL-18), matrix metalloproteinase 9 (MMP-9) and oxidative stress markers—total oxidant status (TOS), total antioxidant capacity (TAC)—evaluated in saliva are modified post-extraction in type 2 diabetes mellitus subjects and whether there is a correlation with HbA1c levels. The aforementioned markers plus HbA1c were investigated in a group of systemically healthy subjects (*n* = 45) and in a type 2 diabetes mellitus group (*n* = 41) before and three months after a tooth extraction. Diabetes patients’ recorded increased levels of OPG, RANKL, TNF-α, MMP-9, IL-18 and TOS compared to controls both pre- and post-extraction. In both study groups, the average OPG, HGF and TAC level recorded an upward trend three months post-extraction. TNF-α registered a statistically significant decrease only in the diabetes group after dental extraction, together with a decrement of mean HbA1c levels in the diabetes group. By plotting the ROC (receiver operating characteristic) curve, at baseline RANKL, TNF-α, IL-18, MMP-9, TOS and OPG were good predictors of HbA1c levels. Post-extraction, there was a significant correlation between HbA1c and oxidative status biomarkers, however the linear regression model indicated the influence of all studied salivary markers in HbA1c determinism, in a considerable proportion. In conclusion, our study demonstrated that several oxidative status markers and proinflammatory biomarkers are modified in the saliva of diabetic patients and they correlate to HbA1c levels, thus being potential indicators of the post-extraction healing status in the oral cavity.

## 1. Introduction

Dental extraction initiates a series of repair processes that involve both hard (alveolar bone) and soft tissue (periodontal ligament, gingiva) remodeling. The healing process consists of a local inflammatory immune reaction caused by the release of multiple molecules that have been described as playing a significant role in bone healing, as demonstrated by the association between anti-inflammatory drugs and delayed bone healing [1]. In-depth knowledge of the healing process, including contour changes caused by bone resorption and remodeling, is essential for optimum wound healing and subsequent bone morphology. Resorption of the alveolar bone may occur prior to tooth extraction due to periodontal disease, periapical pathology, or dental and bone trauma. A traumatic dental extraction can further induce additional bone loss; furthermore, the process of alveolar bone atrophy over time after tooth extraction has also been described [2].

Diabetes mellitus is a metabolic disorder; type 2 diabetes mellitus (T2DM) being the more prevalent form, and the overall burden of this disease is estimated to increase even further in the future [3]. There is substantial evidence to suggest that diabetes is a serious risk factor for many oral pathologies such as periodontal disease, carious disease, painful mouth syndrome, both in terms of progression and severity of these conditions but also regarding subsequent implant-prosthetic rehabilitation possibilities [4]. In addition, pathological conditions such as osteoporosis, uncontrolled diabetes and hypertension have been associated with impaired bone metabolism [5]. Moreover, there is a significant correlation between both HbA1c and salivary glucose levels and patients with diabetes. Thus, blood glucose levels could be monitored by saliva in patients with diabetes [6].

With the development of molecular biology, new controversies have arisen regarding the molecular aspects of bone healing beyond histological studies, especially regarding protein synthesis involved in post-extraction bone healing mechanisms. In this context, salivary diagnosis provided promising biomarkers for monitoring the progression of numerous pathologies due to the simplicity of saliva collection, its non-invasive nature and the accuracy of diagnosis [7]. 

Osteoclastogenesis is controlled by three members of the tumor necrosis factor (TNF) and the TNF receptor superfamily, the kappa B nuclear factor receptor activator (RANK), its ligand (RANKL), and osteoprotegerin (OPG). RANKL binds directly to RANK on the surface of pre-osteoclasts and osteoclasts, and establishes the differentiation of osteoclast progenitors and the activation of mature osteoclasts [8]. Osteoprotegerin is a protein that has a homologous structure to RANK and is therefore a receptor for RANKL. Once the interaction between the ligand and the receptor is interrupted, bone resorption is prevented by reducing osteoclast differentiation. Osteoporosis has been linked to periodontal disease and oral dysbiosis, and some consider the relationship to be bidirectional [9].

Hepatocyte growth factor (HGF) plays important roles in organ development and morphogenesis as a mediator in epithelial–mesenchymal interactions [10]. It has recently been suggested that chronic subclinical inflammation is associated with insulin resistance and precedes the development of type 2 diabetes. Data from a meta-analysis reveals that T2DM (type 2 diabetes mellitus) risk as a whole was solidly correlated with increased levels of inflammatory cytokines such as interleukin (IL) 1β, IL-6, IL-18, C reactive protein (CRP) and tumor necrosis factor-α (TNF-α) [11]. Moreover, it has been shown that elevated levels of TNF-α over a longer period of time may be a predictor of the evolution of diabetic disease [11].

In the oral cavity, matrix metalloproteinases (MMPs) are critical factors in many physiological, but also pathological processes. MMP-9 regulates cell differentiation during osteogenesis and angiogenesis and has been shown to be increased in saliva in oral pathologies such as premalignant lesions and squamous cell carcinoma [12]. 

Oxidative stress, which occurs as a result of excessive production of reactive oxygen species related to the body’s decreased antioxidant capacity, has been investigated in recent years, especially in patients with diabetes and those with various oral pathologies [13]. Some authors have suggested that diabetes is associated with increased oxidative stress, but it is unclear whether oxidative stress is the result of diabetes or a contributing factor, although there is sufficient evidence to highlight its detrimental effect on insulin resistance, especially in obese subjects [14]. An important role in the evolution of many inflammatory diseases including diabetes and several oral pathologies is attributed to reactive oxygen species (ROS). The level or intense activity of ROS cannot be balanced by the antioxidant defense system, and this leads to oxidative stress and ultimately to tissue alteration [15]. The evaluation of oxidative stress is possible by assessing the total antioxidant capacity, the oxidative state and the oxidative stress index. In recent years, a number of studies have shown a strong association between oxidative stress and periodontitis, resulting in a number of metabolites of lipid peroxidation, DNA damage, and protein damage, used to assess tissue destruction [15]. Similarly, changes in the activity of antioxidants in periodontal disease are influenced by systemic conditions [14].

In this context, compound risk assessment that includes clinical, biochemical (serum and saliva derivatives), as well as microbiological risk factors may aid in determining the evolution of diabetic patients by providing an estimation of disease progression or stabilization, both orally and systemically, and could further help in stratifying patients according to their status.

Based on these considerations, in this study we assessed whether the analyzed biomarkers,—osteoprotegerin (OPG), kappa B nuclear factor receptor activator ligand (RANKL), hepatocyte growth factor (HGF), tumor necrosis factor-α (TNF-α), interleukin 18 (IL-18), matrix metalloproteinase 9 (MMP-9) and oxidative stress markers: total oxidant status (TOS), total antioxidant capacity (TAC)—are modified in the saliva of type 2 diabetes mellitus patients compared to healthy individuals pre -and post-dental extraction, and whether they correlate with HbA1c levels.

## 2. Materials and Methods

### 2.1. Study Group

In conducting the research for this case control study, we respected the ethical rules according to the Declaration of Helsinki. Informed consent was obtained from all subjects involved in the study.

The Research Ethics Committee of the University of Medicine and Pharmacy “Grigore T. Popa” Iași approved the study, with the number 20 November 2018. Following the approval of the ethics committee, the subjects were clinically and paraclinically examined.

The inclusion criteria for this study was patients older than 18 years who require at least one tooth extraction. For the diabetes type 2 group, the criteria was patients with a definite diagnosis of type 2 diabetes for at least 3 years (according to the American Diabetes Association), with glycated hemoglobin values between 6.5% and 11%. 

Exclusion criteria: use of antibiotic or anti-inflammatory therapy in the last 3 months, any periodontal or orthodontic treatment in the last 6 months, periodontal disease stage 3 or 4, body mass index (BMI) > 30, psychiatric disorders, pregnant or lactating women, smokers, systemic diseases that may interfere with the immune response, or with bone metabolism (osteoporosis, rheumatoid arthritis, endocrine diseases, etc.) except for diabetes type 2.

The sample size calculation was done by G power analysis considering TNF-α as the primary variable (with an anticipated mean of 4.7 ± 4.1 for the control group and a mean of 8.1 for the diabetes type 2 group, standard deviation 2.3). Taking into account a type 1 error of 0.05 and a type 2 error of 0.05, with a statistical power of 95% we obtained a minimum of 38 patients per group. Considering a 10% drop-out rate the final sample size was 41 patients minimum per group.

The study was carried out between November 2018 and November 2019 and initially included a number of 172 patients. After application of the inclusion and exclusion criteria, 92 patients were selected, of which 6 subjects did not attend the follow up visit and were excluded from the study. The remaining 86 patients with various dental conditions were divided into 2 groups: the control group (C)—45 patients without systemic pathology, and the diabetes mellitus type 2 group (DM)—41 patients. For all diabetes mellitus type 2 subjects included in the study, the therapeutic indications were applied according to the data mentioned in the observation charts plus the post-extraction monitoring that all patients benefited from. Oral hygiene instruction and advice on diet and lifestyle was offered to all patients included in the study. Additionally, a questionnaire on diet and medication was given to all patients and no significant changes were found during the study period.

### 2.2. Saliva Sampling

Approximately 3 mL of unstimulated whole saliva was collected through passively drooling in a sterile centrifuge tube (Sterile Eco ROTH^®^ Tube, 15 mL) from each participant. The sampling was performed between 8.00 and 10.00 am, On an empty stomach, without performing oral hygiene that morning. Following this procedure, in the same morning, venous blood samples were taken from all participants for Hb1A1c (glycated hemoglobin) determination. Afterwards, the samples were immediately placed in a refrigerated box and then stored at −80 °C until analysis of the biochemical markers. 

Saliva and venous blood samples were taken from all patients in two moments: T0: before performing any dental therapeutic operation, and T1: 3 months after tooth extraction.

A number of parameters were determined in saliva, using the ELISA immunenzymatic method: OPG (MyBioSource, San Diego, CA, United States, # MBS175881), RANKL (MyBioSource, San Diego, CA, United States, # MBS268235), HGF (Elabscience, Houston, Texas, United States, Catalog no. E-EL-H0084), TNF-α (Elabscience, Houston, Texas, United States, Catalog no. E-EL-H0109), MMP-9 (Abcam, CA, United Kingdom, ab246539), IL-18 (Abcam, Cambridge, United Kingdom, ab215539), and the spectrophotometric method determined TAC (Rel Assay Diagnostics Kit, Mega Tıp, Gaziantep, Turkey) and TOS (Rel Assay Diagnostics Kit, Mega Tıp, Gaziantep, Turkey), according to the method described by Erel in 2005 [16]. 

All reagents and samples were brought to room temperature and used according to the manufacturer’s instructions.

After the initial sampling (T0), teeth with various pathologies were extracted, according to the diagnosis and the treatment plan elaborated by a dento-alveolar surgeon specialist. After 3 months (T1), the same markers mentioned above were analyzed.

### 2.3. Statistical Analysis

Data were uploaded and processed using the statistical functions in SPSS version 18.0 software (IBM Corp., Armonk, NY, USA) at the 95% significance threshold. Based on the primary indicators, by different statistical procedures of comparison, abstraction and generalization, the derived indicators were obtained. The statistical indicators derived from the ANOVA test were: indicators of the mean value (mean, median, modulus, minimum and maximum values, etc.) and indicators of dispersion (standard error, standard deviation, coefficient of variation). Skewness or Kurtosis tests (−2 < *p* < 2) are tests that can determine whether or not variables are continuous by assessing the normal range of values. Unpaired *t*-tests were used to evaluate differences between the control and diabetes group, and paired-samples *t*-tests were used to evaluate intragroup evolution of mean values. For categorical variables, we applied chi-square and Fisher’s exact test. The receiver operating characteristic (ROC) curve was applied for estimating sensitivity, specificity and c-statistics (area under the curve: AUC). The cut-off was decided to estimate the highest value for the sum of sensitivity and specificity. 

## 3. Results

In Table 1 we illustrate the demographic characteristics of the analyzed study groups, the cause of dental extraction and the baseline HbA1c value.

### Salivary Markers

In Table 2 we present the pre- and post-extraction OPG, RANKL, HGF, TNF-α, IL-18, MMP-9, TOS—total oxidant status, TAC—total antioxidant capacity, and HbA1c values in control and diabetes mellitus patients. According to our results, in both study groups, the mean OPG, OPG/RANKL ratio and TAC level increased significantly three months post-extraction, whereas mean RANKL, MMP-9, IL-18 and TOS level decreased in the same interval. Post-extraction reduction in TNF-α was statistically significant only for the DM group, whereas for HGF we observed an increase at follow-up only in controls. 

DM subjects had significantly higher OPG, RANKL, TNF-α, MMP-9, IL-18, and TOS values compared to controls in both analyzed moments. On the other hand, OPG/RANKL ratio and HGF were higher in the control group in baseline and follow-up; whereas, TAC was higher only at baseline, at follow-up the values being similar.

Mean level of HbA1c was significantly higher in diabetic patients compared to the control group, in both analyzed times (7.98% vs. 5.01%; *p* = 0.001 and 7.62% vs. 5.0 %; *p* = 0.001). In DM subjects, HbA1c mean level recorded statistically significant reductions three months after dental extraction (7.98% vs. 7.62%; *p* = 0.05) (Table 2). Cronbach’s alpha was used for the number of null-hypotheses (*N* = 9) that are tested simultaneously (−0.8960 and alpha was adjusted accordingly to 0.600).

By plotting the ROC curve, at baseline, good predictors of HbA1c values over 6.5% are the following salivary markers: OPG (cut off value 2.77), RANKL (cut off value 15), TNF-α (cut off value 6.02), MMP-9 (cut off value 120), IL-18 (cut off value 198) and TOS (cut off value 17.5) (Table 3).

**Figure 1:**
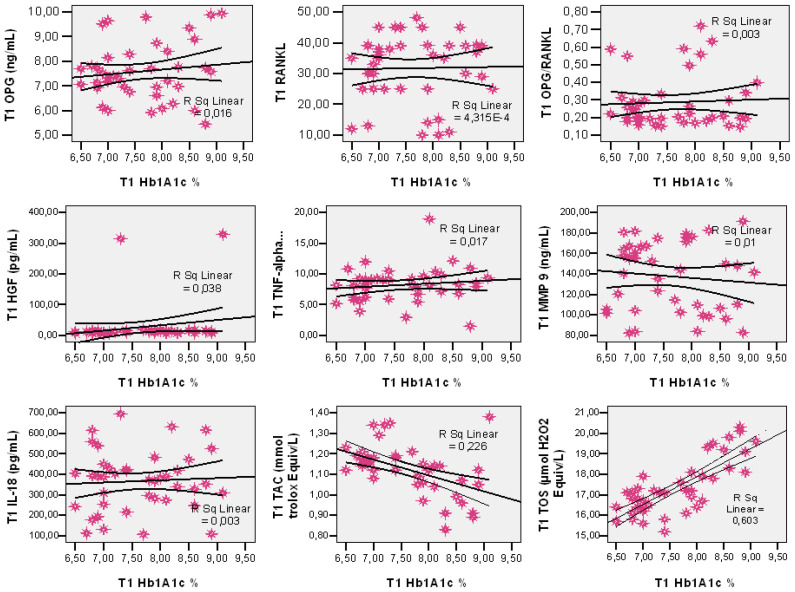
Post-extraction correlation of HbA1c with salivary markers (OPG, RANKL, OPG/RANKL, HGF, TNF-α, MMP-9, IL-18, TOS and TAC).

In the DM group, three months after dental extraction, the individual values of HbA1c recorded the following correlations with salivary markers: an indirect correlation, moderate in intensity, was observed with TAC (r = −0.475; R = 0.226; *p* = 0.002) and a direct correlation, moderate in intensity, with TOS (r = +0.777; R = 0.603; *p* = 0.001) (Figure 1). There was a direct correlation, reduced in intensity, with OPG, HGF and TNF-α (r = +0.128; R = 0.016; *p* = 0.424; r = +0.194; R = 0.038; *p* = 0.224 and r = +0.131; R = 0.017; *p* = 0.415). On the other hand, HbA1c and RANKL, OPG/RANKL, MMP-9 and IL-18 are apparently independent parameters (r = +0.052; R = 0.0004; *p* = 0.746; r = +0.021; R = 0.003; *p* = 0.897; r = −0.098; R = 0.01; *p* = 0.540; and r = +0.057; R = 0.003; *p* = 0.725) (Figure 1). 

**Figure 2:**
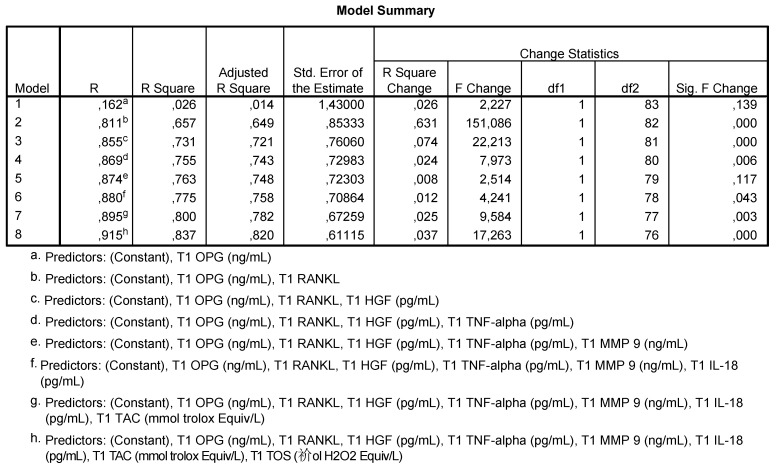
Multivariate analysis; HbA1c post-extraction.

We used multivariate analysis to quantify the intensity and direction of influence of the correlation between the analyzed factors (Figure 2). Multiple linear regression establishes the predictive efficiency of detected salivary markers (independent variables) on the HbA1c dependent variable post-extraction.

Models that suggest that some salivary parameters may be good predictors for the evolution of post-extraction HbA1c are the following (Figure 2):

- Model 2 shows that 65.7% of the HbA1c values are correlated to OPG and RANKL levels;
- Model 3 shows that 73.1% of HbA1c values are correlated to OPG, RANKL and HGF levels;
- Model 4 shows that 75.5% of HbA1c values are correlated to OPG, RANKL, HGF and TNF-α levels;
- Model 5 shows that 77.5% of HbA1c values are correlated to the levels of OPG, RANKL, HGF, TNF-α, MMP-9 and IL-18;
- Model 6 shows that 80% of HbA1c values are correlated to the levels of OPG, RANKL, HGF, TNF-α, MMP-9, IL-18 and TAC;
- Model h shows that 83.7% of HbA1c are correlated to the levels of all analyzed salivary markers.

The regression line resulting from the multivariate analysis was as follows:

y = 2.07 + 0.066 OPG + 0.013 RANKL + 0.001 HGF + 0.061 TNF-α—0.001 MMP-9 + +0.001 IL-18—0.037 TAC + 0.197 TOS, highlighting direct correlations with all salivary markers except MMP-9 and TAC.

## 4. Discussion

When considering the ease of collection, non-invasive character and accuracy of diagnosis, saliva biomarkers are promising sources for monitoring the onset and progression of numerous oral and systemic pathologies.

Our results of the OPG evaluation are in accordance with several studies that analyzed the level of OPG in blood/saliva or crevicular fluid. Valentini et al. showed that diabetics have higher plasma OPG concentrations and a lower RANKL/OPG ratio compared to non-diabetics, with OPG levels directly correlating with HbA1c levels [17]. In order to have a more complete image of the bone remodeling dynamics, OPG/RANKL ratio was also analyzed, and we observed that the initial value was significantly higher in DM group compared to C; post-extraction, the value was reduced for both groups, especially in the systemically affected group. These values indicate a modified status in terms of bone turnover in diabetic patients. Moreover, the elimination of local pro-inflammatory factors through the treatment of oral pathologies has led to an improvement in the values of these markers in this systemically affected group with multiple possible implications on homeostasis. Therefore, diabetes appears to be an important factor influencing the local OPG/RANKL ratio. 

Using molecular techniques (semiquantitative PCR chain amplification), some studies analyzed gene expression in gingival tissue in patients with chronic periodontitis, reporting that diabetic status was not a significant factor in changes in RANKL expression, but OPG expression showed a decreasing trend when compared to non-diabetics [18]. In our study, both RANKL and OPG showed increased values when compared to non-diabetics both pre- and post-extraction. Another study analyzing these variations in gingival crevicular fluid highlighted that RANKL levels and RANKL/OPG ratio is higher in poorly controlled diabetics with chronic periodontitis, compared to healthy individuals but also subjects with diabetes with optimal metabolic control and periodontitis, and this ratio was not significantly affected by periodontal therapy [19]. Thus, our study strengthens the belief that the RANKL/OPG ratio is negatively influenced in diabetes, especially in subjects with poor glycemic control.

In our study, mean HGF level increased in both groups after dental extraction. The role of HGF in different organs and structures is still being investigated and further research is needed in this particular aspect. A study that evaluates the links between HGF and insulin resistance highlights the beneficial effects of HGF in subjects with metabolic syndrome [20]. That being said, there is evidence that elevated HGF levels is correlated with the manifestation of renal dysfunction, manifested through nephropathy [21]. Given that HGF significantly increases glucose metabolism and transport to myocytes and adipocytes, HGF could be used as a therapeutic target in the treatment of insulin resistance [22]. 

The role of TNF-α as a major risk factor for type 2 diabetes and insulin resistance has been debated in several studies linking obesity with diabetic disease [23,24]. A recent study showed that elevated salivary levels of C-reactive protein and TNF-α have been significantly associated with type 2 diabetes [25]. Several studies have indicated elevated levels of TNF-α in patients with periodontitis, peri-implant disease, oral squamous cell carcinoma, and oral candidiasis [26,27,28]. In our study we obtained significantly increased salivary TNF-α values in diabetics compared to controls at baseline and at follow-up. Furthermore, a statistically significant reduction after dental extraction occurred only in diabetic patients. Interestingly, several studies have attributed changes in vascular permeability, especially those occurring in diabetes, to TNF-α overexpression [11,29]. This could cause excess edema after dental extraction and delay wound healing.

Numerous studies in the literature have shown that MMPs (such as MMP-2, MMP-7, MMP-8, MMP-9, MMP-14) register altered values in patients with DM (types 1 and 2) and this seems to contribute to several complications of diabetes [30,31]. In our study, diabetic patients recorded higher values compared to controls. Our results are similar to those expressed in the study by Yu et al., in which subjects that had at least one of the characteristics of metabolic syndrome (central obesity, low HDL cholesterol, high blood pressure, high fasting blood glucose) also presented elevated MMP-9 levels when compared to subjects without metabolic syndrome [32]. Collectively, these data suggest that altered remodeling of the extracellular matrix could delay subsequent bone formation after tooth extraction.

In the present study, in diabetes patients, IL-18 was higher in value both pre- and post-extraction when compared to systemically healthy patients, which confirms the increased inflammatory load for these individuals. A study found similar elevated values measured in 527 subjects with type 2 diabetes and 1698 non-diabetic subjects. The authors observed elevated levels of IL-18 as being associated with a significantly increased type 2 diabetes risk, after adjusting for age, gender, BMI (body mass index), systolic blood pressure, total cholesterol and HDL ratio, physical activity, alcohol intake, smoking and parents’ history of diabetes. In addition, the risk of developing type 2 diabetes was highest among subjects with elevated IL-18 and CRP or IL-18 and IL-6, respectively [33]. In both our analyzed groups, IL-18 values decreased after dental extraction. A study that evaluated this cytokine in healthy vs. periodontally involved patients observed a similar upward trend in periodontitis subjects; however, these values significantly decreased after nonsurgical periodontal therapy [34].

In a recent study analyzing levels of the following antioxidant and oxidative stress markers—MDA (malondialdehyde), TOS, TAC, SOD (superoxide dismutase), glutathione peroxidase and IL-6—in 30 patients newly diagnosed with type 2 diabetes and 30 systemically healthy patients, MDA, TOS and IL-6 values were significantly up marked in diabetic patients. However, TAC and SOD were significantly decreased in the same group. Moreover, there was a significant positive correlation between IL-6 with MDA and TOS, and a negative correlation between IL-6 and SOD activity [35]. These results are similar to our own and they emphasize the important oxidative stress imbalance caused by diabetes.

There is sufficient evidence to suggest that periodontal inflammation is one of the main sources of reactive oxygen species in the oral cavity, accrediting the concept that periodontal tissue damage is the result of an imbalance between bacterial flora and host defense mechanisms, including the release of reactive oxygen species by neutrophils and other immune cells. This hypothesis is current and attracts growing interest from the scientific community [36,37,38,39]. In gingival fluid, saliva and peripheral blood of patients with chronic and/or aggressive periodontitis, low concentrations of total antioxidant capacity were observed when compared to healthy subjects [40,41]. In addition, recent research suggests that melatonin (a hormone that possesses antioxidant qualities) can be seen as a useful diagnostic marker in periodontitis, and can be used for the differential diagnosis of chronic and aggressive periodontitis [41]. Furthermore, a recent study that evaluated non-surgical periodontal treatment and melatonin supplementation in type 2 diabetes subjects, found additional improvements of HbA1c levels and periodontal disease severity in patients treated with SRP and melatonin vs. SRP alone [42]. 

In our study, the mean level of TOS was significantly higher in diabetic patients compared to the control group, in both analyzed moments; moreover, in both groups it decreased post-extraction. Total antioxidant capacity was significantly lower in diabetic patients, only at baseline; afterwards there was a higher average level in both study groups, revealing an increase in antioxidant capacity consequent to the elimination of inflammatory sources and generators of oxidative stress in the oral cavity. Data similar to ours was reported by a recent study that compared the salivary levels of some markers of oxidative stress: TOS, MDA, the level of thiol groups and total antioxidant capacity, uric acid, peroxidase and catalase in subjects with diabetes. The authors reported low levels of TAC and salivary catalase, while MDA and TOS were significantly higher in patients with type 2 diabetes compared to healthy controls [43]. In another study that analyzed the total oxidative and antioxidant activity in gingival crevicular fluid and saliva in patients with periodontitis by calculating the oxidative stress index (OSI), the authors evaluated the intensity of redox disorders. Thus, in unstimulated/stimulated saliva, as well as gingival crevicular fluid of the study group, the researchers reported significantly higher OSI and TOS values, as well as a lower antioxidant capacity. The examined parameters were correlated with the clinical condition of the periodontium, which indicates the exacerbation of the inflammatory process. However, comparison of TAC, TOS and OSI levels could not prove relevant to differentiate between individual degrees of severity of periodontitis [44].

The scientific community is increasingly promoting the objective discovery of quantifiable biomarkers in oral fluids that can truly reflect the oral pathophysiological condition, with the possibility of improving sensitivity and specificity in the early detection and monitoring of oral pathologies [45]. 

In the present study, in the DM group, glycated hemoglobin values, a monitor of blood glucose variation over a period corresponding to the erythrocyte life cycle, varied at the beginning of the study from 6.70% to 9.70%, highlighting that all patients in this group had a level of HbA1c over 6.50%. Moreover, 22% were unbalanced diabetic patients, with an HbA1c level over 9%. In the DM group, we observed a decreased HbA1c value after dental extraction, due to a better oral status by removing the infected tooth, results similar to those obtained by a randomized clinical trial [46]. Furthermore, Sundar, in 2018, concluded that nonsurgical periodontal therapy is correlated with an important decrease in HbA1c levels in T2DM patients with periodontitis [47]. 

Variations in the severity of inflammation lead to differences in the variety and concentrations of inflammatory markers. According to one study, AUC had the largest values for MMP-8, myeloperoxidase and MMP-9 in subjects with periodontal disease [48], the sensitivity to diagnosis being similar to the results obtained in the present study. Gursoy et al. proposed a new diagnostic approach by using three different salivary markers (*Porphyromonas gingivalis*, IL-1β and MMP-8) for which both ROC, logistic regression analysis and a cumulative risk score (CRS) were calculated and analyzed. Each biomarker had an association with periodontitis at a different level, and the association increased considerably by using cumulative risk scores, demonstrated in the ROC analysis, where the AUC value increased from 0.694–0.710 to 0.766 [49]. Similar in intent to our study, the authors propose this model as a non-invasive tool for risk categorization, especially in wide population studies. Corroborating our results with those in the literature, we can estimate that the significant reduction in total antioxidant capacity and increased total oxidative stress is most likely due to the chronic inflammatory process, aggravated even more by the presence of diabetes. Such a condition can predispose to oxidative damage of proteins, lipids and genetic material, and can cause the progressive destruction of dental supporting tissues.

To our knowledge, this is the first study to correlate salivary markers of oral impairment and corroborate them with glycated hemoglobin values, a marker of diabetes severity, in an attempt to assess a patient’s prognosis regarding oral surgery. We also sought to select potential biomarkers that could provide a complete clinical image by establishing a fast and non-invasive “chair-side” test that can be performed in saliva to identify subjects at high risk of developing complications. 

Nonetheless, these results must be interpreted with caution and a number of limitations should be borne in mind. Prior research studies that are relevant to this study are limited and the comparative issues that are needed to be made are difficult, especially in the complex context of diabetic patients. Furthermore, several other factors have to be considered such as BMI, duration of disease, other existing comorbidities and lifestyle. Supplementary data on the periodontal status and interactions must be added in order to clarify certain aspects of the complex interactions in the oral microenvironment. Another limitation is the fact that our study was performed on Caucasian patients, thus limiting the data only to this category.

## 5. Conclusions

Oral health is an indicator of the general health of the body, and this can help guide a treatment plan that will provide the most benefits to the patient. We have analyzed potential biomarkers that could provide a complete clinical picture by establishing a rapid and non-invasive test to identify subjects at high risk of developing complications.

At baseline RANKL, TNF-α, IL-18, MMP-9, TOS and OPG were good predictors of HbA1c levels. Post-extraction, there was a significant correlation between HbA1c and oxidative status biomarkers, however the linear regression model indicated the influence of all studied salivary markers in HbA1c determinism, in a considerable proportion.

Our study demonstrated that several oxidative status markers and proinflammatory biomarkers are modified in the saliva of diabetic patients and they could be used in the evaluation of wound healing in the oral cavity. 

## Figures and Tables

**Table 1:** Descriptive data for the study participants.

Parameters	All Cases (*n* = 86)	Control Group (*n* = 45)	Diabetes Mellitus Group (*n* = 41)	*p*-Value
Age (years), mean ± SD	52.60 ± 12.65	51.02 ± 13.56	54.34 ± 11.50	0.226 ^(a)^
(min–max)	(28–78)	(28–78)	(34–78)	
Gender				0.814 ^(b)^
Male *n*(%)	41 (47.7%)	22 (48.9%)	19 (46.3%)	
Female *n*(%)	45 (52.3%)	23 (51.1%)	22 (53.7%)	
Area				0.782 ^(b)^
Urban	49 (57.0%)	25 (55.6%)	24 (58.5%)	
Rural	37 (43.0%)	20 (44.4%)	17 (41.5%)	
HbA1c (%), mean ± SD	4.43 ± 1.65	5.01 ± 0.43	7.98 ± 0.91	0.001 ^(a)^
(min–max)	(4.20–9.70)	(4.20–5.80)	(6.70–9.70)	
Cause of extraction				0.019 ^(c)^
Root rest (carious disease/Periapical lesion)	26 (30.2%)	13 (28.9%)	13 (31.7%)	
Periodontal disease	41 (47.7%)	18 (40.0%)	23 (56.1%)	
Dental inclusion	19 (22.1%)	14 (31.1%)	5 (12.2%)	

SD—standard deviation^; (^^a)^ Student’s *t*-test; ^(b^^)^ Fisher’s exact test; ^(c^^)^ Chi-square test likelihood-ratio, HbA1c—glycated hemoglobin. *p* < 0.05 is considered statistically significant.

**Table 2:** Pre- and post-extraction OPG, RANKL, HGF, TNF-α, IL-18, MMP-9, TOS—total oxidant status, TAC—total antioxidant capacity, and HbA1c values in control and diabetes mellitus patients.

	Parameters	Lot C		Lot DM	
		T0	T1	T0	T1
OPG	Mean	2.08	6.73	3.79 (**)	7.58 (*)
	Std. Dev.	1.36	2.49	0.84	1.17
	Paired-Samples *t*-test (*p*-values)	0.001		0.001	
	Skewness test	0.446	0.452	−0.699	0.463
RANKL	Mean	8.02	3.02	36.70 (**)	31.70 (**)
	Std. Dev.	2.86	2.80	10.93	10.92
	Paired-Samples *t*-test (*p*-values)	0.001		0.045	
	Skewness test	1.376	1.376	−0.704	−0.704
OPG/ RANKL	Mean	0.29	5.47	0.12 (**)	0.29 (**)
	Std. Dev.	0.23	0.06	6.12	0.15
	Paired-Samples *t*-test (*p*-values)	0.001		0.001	
	Skewness test	0.833	1.610	1.938	1.465
HGF	Mean	211.76	874.94	13.29 (**)	26.38 (**)
	Std. Dev.	106.24	332.29	12.10	27.78
	Paired-Samples *t*-test (*p*-values)	0.001		0.181	
	Skewness test	0.924	1.004	5.204	4.332
TNF-α	Mean	4.81	4.57	9.69 (**)	8.28 (**)
	Std. Dev.	2.10	2.12	2.97	2.82
	Paired-Samples *t*-test (*p*-values)	0.616		0.001	
	Skewness test	0.021	0.442	0.168	0.901
MMP-9	Mean	91.40	70.78	160.90 (**)	137.63 (**)
	Std. Dev.	44.82	36.88	37.21	33.90
	Paired-Samples *t*-test (*p*-values)	0.001		0.001	
	Skewness test	0.605	0.695	1.040	−0.185
IL-18	Mean	223.36	147.23	480.25 (**)	366.57 (**)
	Std. Dev.	76.95	42.41	190.17	146.33
	Paired-Samples *t*-test (*p*-values)	0.001		0.001	
	Skewness test	0.110	0.456	−0.117	0.151
TOS	Mean	15.40	13.51	18.92 (**)	17.38 (**)
	Std. Dev.	2.62	2.16	1.75	1.33
	Paired-Samples *t*-test (*p*-values)	0.001		0.001	
	Skewness test	−0.268	−0.017	0.915	0.672
TAC	Mean	0.83	1.12	0.73 (**)	1.12 (ns)
	Std. Dev.	0.19	0.13	0.11	0.12
	Paired-Samples *t*-test (*p*-values)	0.001		0.001	
	Skewness test	0.645	−0.151	0.739	−0.163
HbA1c	Mean	5.01	5.00	7.98 (**)	7.62 (**)
	Std. Dev.	0.43	0.29	0.91	0.76
	Paired-Samples *t*-test (*p*-values)	0.697		0.050	
	Skewness test	0.121	−0.404	0.351	0.340
	% normal values	91.1	100.0	-	-
	% prediabetes	8.9	-	-	-
	% DM	-	-	100.0	100.0
	% uncontrolled DM	-	-	22.0	2.4

Lot C—Control group; Lot DM—Diabetes mellitus group; OPG—osteoprotegerin; RANKL—receptor activator of nuclear factor kappa-Β ligand; HGF—hepatocyte growth factor; TNF-α—tumor necrosis factor α; MMP-9—matrix metalloproteinase 9; IL-18—interleukin 18; TOS—total oxidant status, TAC—total antioxidant capacity; HbA1c—glycated hemoglobin. *p* < 0.05 is considered statistically significant. Student *t*-test in DM vs. C: (**) *p* < 0.001; (*) *p* < 0.05, (ns) not significance. Skewness test (−2 < *p* < 2) suggests that the value series of salivary parameters had normal distributions.

**Table 3:** ROC curve. Prediction of salivary markers in the determinism of HbA1c > 6.5%.

Salivary Parameters	Cut off	Se	Sp	AUC	Std. Error	*p*	Confidence Interval 95%
OPG (ng/mL)	2.77	84.6%	65.9%	0.833	0.044	0.001	0.747–0.920
RANKL	15	99.0%	97.6%	0.999	0.001	0.001	0.996–1.002
OPG/RANKL	-	-	-	0.204	0.052	0.001	0.103–0.305
HGF (pg/mL)	-	-	-	0.001	0.001	0.001	0.001–0.003
TNF-α (pg/mL)	6.02	92.5%	65.9%	0.907	0.032	0.001	0.844–0.969
MMP-9 (ng/mL)	120	92.3%	80.5%	0.899	0.037	0.001	0.826–0.972
IL-18 (pg/mL)	198	92.3%	54.0%	0.901	0.039	0.001	0.825–0.977
TAC (mmol trolox Equiv/L)	-	-	-	0.339	0.061	0.013	0.219–0.458
TOS (µmol H2O2 Equiv/L)	17.5	87.2%	75.6%	0.876	0.039	0.001	0.800–0.951

AUC—area under the curve; Se—sensitivity; Sp—specificity; OPG—osteoprotegerin; RANKL—receptor activator of nuclear factor kappa-Β ligand; HGF—hepatocyte growth factor; TNF-α—tumor necrosis factor α; MMP-9—matrix metalloproteinase 9; IL-18—interleukin 18; TAC—otal antioxidant capacity; TOS—total oxidant status, HbA1c—glycated hemoglobin. *p* < 0.05 is considered statistically significant.

## Data Availability

The data presented in this study are available in this manuscript.
